# Chromatin accessibility-based characterisation of brain gene regulatory networks in three distinct honey bee polyphenisms

**DOI:** 10.1093/nar/gkac992

**Published:** 2022-11-04

**Authors:** Robert Lowe, Marek Wojciechowski, Nancy Ellis, Paul J Hurd

**Affiliations:** RER Consultants, 28 Worbeck Road, London SE20 7SW, UK; School of Biological and Behavioural Sciences, Queen Mary University of London, Mile End Road, London E1 4NS, UK; School of Biological and Behavioural Sciences, Queen Mary University of London, Mile End Road, London E1 4NS, UK; School of Biological and Behavioural Sciences, Queen Mary University of London, Mile End Road, London E1 4NS, UK

## Abstract

The honey bee genome has the capacity to produce three phenotypically distinct organisms (two diploid female castes: queen and worker, and a haploid male drone). Previous studies have implicated metabolic flux acting via epigenetic regulation in directing nutrition-driven phenotypic plasticity in the honey bee. However, the *cis*-acting DNA regulatory elements that establish tissue and polyphenism -specific epigenomes and gene expression programmes, remain unclear. Using a high resolution multiomic approach including assay for transposase-accessible chromatin by sequencing (ATAC-seq), RNA-seq and ChIP-seq, we produce the first genome-wide maps of the regulatory landscape across all three adult honey bee phenotypes identifying > 5000 regulatory regions in queen, 7500 in worker and 6500 in drone, with the vast majority of these sites located within intronic regions. These regions are defined by positive enrichment of H3K27ac and depletion of H3K4me3 and show a positive correlation with gene expression. Using ATAC-seq footprinting we determine queen, worker and drone -specific transcription factor occupancy and uncover novel phenotype-specific regulatory networks identifying two key nuclear receptors that have previously been implicated in caste-determination and adult behavioural maturation in honey bees; ecdysone receptor and ultraspiracle. Collectively, this study provides novel insights into key gene regulatory networks that are associated with these distinct polyphenisms in the honey bee.

## INTRODUCTION

The eusocial insect species of *Hymenoptera* (all ants, and some bees and wasps) are characterised by remarkable polyphenism whereby multiple distinct adult phenotypes emerge from a single genome in response to environmental cues (1). At a mechanistic level, epigenetic systems have been implicated in mediating the integration of environmental cues with transcriptional programmes that result in, and maintain, alternate developmental and behavioural outcomes from the same insect genome (2–5). Honey bees (*Apis mellifera*) are haplodiploid eusocial organisms that live in complex societies comprising tens of thousands of individuals. Each colony contains two main diploid female castes: a single queen who is specialised for reproduction and thousands of sterile female worker bees. A third phenotypic outcome, which develops from unfertilized eggs, is a haploid male drone. The key feature in the establishment of these three post-embryonic developmental trajectories is differential nutrition, which results in morphologically, physiologically, and behaviourally distinct but genetically almost identical organisms (6–11). Significantly, differential feeding continues throughout adulthood (7,10) and may be important in maintaining certain aspects of phenotypic identity such as longevity (12,13). The honey bee genome therefore exemplifies environmentally driven phenotypic plasticity, where diet dictates the ability of different phenotypes to arise from a single genome. The molecular mechanisms responsible for polyphenism in the honey bee are yet to be fully elucidated but multiple levels of epigenetic regulation have been implicated in establishing and maintaining the two female castes. Important work by Maleszka and colleagues has established an important role for DNA methylation in determining queen versus worker developmental trajectories, possibly via modulating gene activities like conditional exon choice (14–16). More recently, RNA-based epigenetic mechanisms including caste-specific microRNAs (17) and *N^6^*-methyladenosine mRNA modification (18) have also been implicated in caste differentiation. Finally, our proteomic and epigenomic approaches, have also demonstrated that honey bee histone proteins are extensively post-translationally modified and that at a crucial developmental stage when canalisation is irreversible, have caste-specific signatures that correlate with caste-specific transcriptional programmes (19,20). Taken together, these and numerous other mRNA profiling studies, have demonstrated that caste determination is a gradual, threshold-based process that requires coordinated actions of hundreds of genes in order to execute a desired phenotypic outcome (21–24).

Cell and tissue -specific transcriptional programmes are orchestrated by sequence-specific DNA-binding proteins such as transcription factors (TF), that interact with *cis*-acting DNA regulatory elements (CREs) in order to establish the activity of target promoters, determine persistent epigenetic patterns and direct 3D genome conformation (25,26). For the vast majority of TFs, the ability to access and bind cognate DNA sequence motifs is determined by nucleosome occupancy and higher-order chromatin organisation. The first genome-wide surveys of chromatin accessibility using DNase I hypersensitive site sequencing (DNase-seq), revealed that in the human genome > 90% of regions bound by TFs were exclusively in nucleosome-free regions (27). Since then, numerous additional experimental approaches have been developed in order to determine genome-wide chromatin accessibility and to identify putative CREs such as enhancers and insulators (28). Given the central role of nucleosomes in regulating accessibility to genomic DNA, techniques employing micrococcal nuclease sequencing (MNase-seq) (29) and assay for transposase-accessible chromatin by sequencing (ATAC-seq) (30,31) have proved to be particularly informative in characterising CREs. Moreover, when combined with methods to determine local histone modification states such as chromatin immunoprecipitation sequencing (ChIP-seq), characteristic features of CREs have been revealed, allowing the subsequent annotation of further functional DNA regulatory elements across numerous different cell types and genomes. For example, *cis*-regulatory functional DNA elements such as enhancers are enriched in transcription factor binding sites (TFBS) and characterised by specific chromatin states which reflect activity in a given genomic context. Active enhancers are typically characterised by nucleosome-free regions flanked by various combinations of H3K4me1, H3K4me3 and H3K27ac modified nucleosomes, neutral/intermediate enhancers by H3K4me1, and poised enhancers with H3K4me1 and H3K27me3 (32–35). Therefore, the landscape of chromatin organisation in a cell is a critical determinant of genome function and in particular, the modulation of TF binding and transcriptional regulation. Understanding how these regulatory DNA regions function and identifying the *cis*-acting factors and environmental signals that regulate them, is a fundamental challenge in cell and developmental biology (28,36).

Here, using high-resolution ATAC-seq, we report the first genome-wide analysis of chromatin accessibility across the honey bee genome in the brains of all three adult polyphenisms and identify thousands of phenotype-specific differences. Furthermore, using a multomic approach and integrating H3K4me1, H3K4me3, H3K27ac and H3K27me3 ChIP-seq and RNA-seq, we also reveal the first *cis*-regulatory DNA sequence maps and the relationship to gene expression and chromatin modification states. Finally, we identify TF binding site occupancy across all adult polyphenisms and determine TF:TF regulatory networks associated with contrasting adult honey bee phenotypes.

## MATERIALS AND METHODS

### Adult honey bee collection and dissection

Frames containing capped worker (W) or drone (D) honey bees of known developmental age, were removed from healthy hives 24 h prior to eclosion, caged and placed in an incubator at 35°C and ∼80% humidity. To obtain adult queens (Q), first instar larvae were grafted into queen cups using standard queen rearing techniques (37). Twenty-four hours prior to eclosure, queen cells were deposited into roller cages and placed in an incubator at 35°C and ∼80% humidity. All newly emerged adult honey bees originated from the same mother queen and were collected within an hour of eclosure. After removal from the incubator, the brains of newly-eclosed adult Q, W and D honey bees were immediately dissected in ice-cold PBS containing cOmplete™ EDTA-free Protease Inhibitor Cocktail (Roche). Care was taken to completely remove the hypopharyngeal glands from workers, and optic lobes were removed from all brain dissections in order to reduce tissue complexity.

### ATAC-seq

Biological replicate pools of five dissected brains were placed in 200 μl of PBS containing cOmplete™ EDTA-free Protease Inhibitor Cocktail. Pools of dissected brains were then gently homogenised by hand with a plastic pestle and centrifuged (900 × g for 5 min). Supernatants were removed and pellets resuspended in 1 ml of lysis buffer (10 mM Tris–HCl pH 7.5/10 mM NaCl/3 mM MgCl_2_/0.2% (v/v) IGEPAL CA-630/cOmplete™ EDTA-free Protease Inhibitor Cocktail) by pipetting for 30 seconds. 50 μl aliquots were removed, centrifuged (900 × g for 5 min) and pellets resuspended in 50 μl of tagmentation buffer (40 mM Tris–HCl pH 7.5/10 mM MgCl_2_/20% (v/v) dimethylformamide). 0.75 μl of Tn5 transposase was added (gift from Dr Chema Martín-Durán, Queen Mary University of London) and tagmentation proceeded for 30 min at 37°C. All transposed samples were purified using a DNA Clean & Concentrator-5 kit, using the binding buffer ratio for small PCR products (Zymo Research). Following purification, ATAC-seq library amplification was carried out with 2× Q5 Polymerase Master Mix and 1.25 μM of Nextera primers (30). The optimal cycle number was determined using quantitative polymerase chain reaction (qPCR) as originally described (30). ATAC-seq libraries were sequenced at Novogene (Cambridge Sequencing Centre, UK) on the Novaseq S4 platform to obtain 96–170M 150 bp paired-end sequencing reads per sample (Supplemental Table S1).

### Chromatin isolation, immunoprecipitation and ChIP-seq

Biological replicate pools of five dissected brains were placed in 200 μl of PBS containing cOmplete™ EDTA-free Protease Inhibitor Cocktail and then gently homogenised by hand with a plastic pestle and centrifuged (900 × g for 5 min). Supernatants were then removed and pellets resuspended in lysis buffer and chromatin was extracted from adult brains, cross-linked, sonicated and immunoprecipitated as described before (20). Antibodies (H3K4me3 [Active Motif, 39159]; H3K4me1 [Abcam, ab8895]; H3K27ac [Active Motif, 39133]; H3K27me3 [Millipore, 07449]) were added according to the manufacturer's instructions and samples were incubated overnight on a rotator mixer at 4°C. DNA was purified with a DNA Clean & Concentrator-5 kit (Zymo Research). NEBNext Ultra II DNA Library Prep Kit for Illumina (New England Biolabs; NEB) was used to make sequencing libraries from 0.5 to 1 ng of DNA following manufacturer's instructions. ChIP-seq libraries were sequenced at Novogene (Cambridge Sequencing Centre, UK) on the Novaseq S4 platform to obtain 83–167M 150 bp paired-end sequencing reads per sample (Supplemental Table S1).

### RNA isolation and RNA-seq library preparation

Biological replicate pools of five dissected adult brains were snap frozen in liquid nitrogen. Total RNA was isolated using the TRIzol method, followed by a RNA Clean-up & Concentration kit (Zymo Research). mRNA was isolated with poly(A) mRNA Magnetic Isolation Module (NEB) from 1 μg of total RNA. RNA-seq libraries were constructed using the NEBNext Ultra Directional RNA Library Prep Kit for Illumina (NEB) following the manufacturer's instructions. RNA-seq libraries were sequenced at Novogene (Cambridge Sequencing Centre, UK) on the Novaseq S4 platform to obtain 17–100M 150 bp paired-end sequencing reads per sample (Supplemental Table S1).

### Data analysis

The genome assembly HAv3.1 (GCF_003254395.2) was downloaded from the NCBI and indexed using BWA-MEM (v0.7.17) (38).

### ATAC-seq data analysis

ATAC-seq samples were mapped to the indexed genome using BWA with default parameters. Detailed mapping statistics for each sample is available in Supplemental Table S1. Reads were split into three categories based on fragment length (0–100 bp, NFR; 150–247 bp, mono-nucleosome; 315–473 bp di-nucleosome) (30). Peaks were called using MACS2 (v2.2.7.1) (39) with the genome size set to 2.7 × 10^8^ bp. Differential peak analysis was performed using DiffBind (v3.0.3) (http://bioconductor.org/packages/release/bioc/vignettes/DiffBind/inst/doc/DiffBind.pdf). Heatmaps showing ATAC-seq peak enrichment were created using DeepTools (v3.5.0) (40). TOBIAS (v0.12.6) (41) was used to perform TF binding analysis by first running footprinting analysis with the appropriate bias corrected data and then matching footprints to motifs using the CORE Insects Non-redundant motifs from JASPAR2020 (42). Given that TFBSs are highly conserved (43,44), the CORE Insect motifs mostly contain motifs discovered in *Drosophila melanogaster*. TF–TF networks were then created using the CreateNetwork tool provided by TOBIAS (41).

### ChIP-seq data analysis

ChIP-seq samples were mapped to the indexed genome using BWA with default parameters. Detailed mapping statistics for each sample are available in Supplemental Table S1. Peaks were called using MACS2 (v2.2.7.1) (39) with genome size set to 2.7 × 10^8^ bp and then differential peak analysis was performed using DiffBind (v3.0.3) (http://bioconductor.org/packages/release/bioc/vignettes/DiffBind/inst/doc/DiffBind.pdf). To calculate the overlap between peaks, the R package GenomicRanges was used (45). First, only peaks which overlapped with a peak called in the replicate peak were kept and these were merged to form a consensus peak set. Then peaks were overlapped with each of the other histone PTMs to determine if they were unique to that histone PTM, or unique to two or three histone PTMs. Heatmaps showing histone PTM enrichment were created using DeepTools (v3.5.0) (40).

### RNA-seq data analysis

The cDNA of reference transcripts and ncRNAs were downloaded from EnsemblMetazoa in FASTA format using genome version GCF_003254395.2. These two FASTA files were concatenated and supercontigs were removed using linux command grep with the following string ‘supercontig|‘‘$genome_version’’:[^1–9XMY]’. Kallisto (46) was used to build an index for further mapping using default parameters. Each sample's FASTQ file was mapped using Kallisto quant with default parameters except for increasing the number of bootstrap samples to 100 and setting the strand specific nature of the reads using parameters ‘-b 100 –rf-stranded’. Detailed mapping statistics for each sample is available in Supplemental Table S1. To determine differential expression, the resulting files from the mapping were used with the R program sleuth (47). Default parameters were used throughout analysis. Sleuth uses a likelihood ratio test and hence we tested for those genes whose abundance is significantly better explained when caste is included in the model compared to a reduced model in which a single parameter is fitted for each gene.

### Gene Ontology analysis

Gene Ontology (GO) terms were downloaded from HymenopteraMine [http://hymenopteramine-v15.rnet.missouri.edu/hymenopteramine/begin.do]. GO analysis was performed using the R package; topGO (https://bioconductor.org/packages/release/bioc/html/topGO.html).

## RESULTS

### Chromatin accessibility in *A. mellifera* is localised mainly to intronic regions and correlates with active transcription

**Figure 1.**
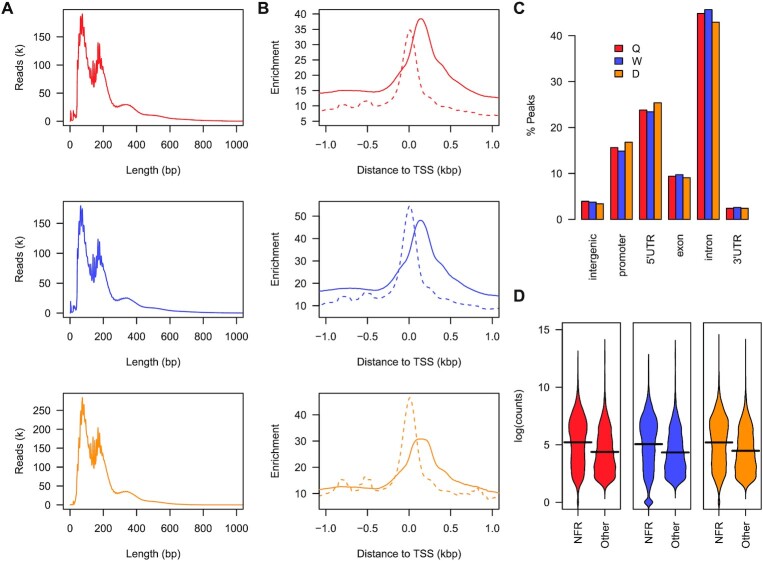
ATAC-seq identifies accessible chromatin regions and nucleosome occupancy across the *A. mellifera* genome. (**A**) ATAC-seq fragment size distribution corresponding to Q (red), W (blue) and D (orange). (**B**) Plots of the average ATAC-seq enrichment around the transcriptional start sites (±1 kb) of genes profiled across Q (red), W (blue) and D (orange). Mono-nucleosomes (solid) and nucleosome-free regions (dashed) are indicated. (**C**) A bar plot showing the percentage of nucleosome-free regions that overlap with an annotated genomic region in Q, W and D. (**D**) The expression distribution of transcripts (measured as log (normalised counts)) for genes containing at least one nucleosome-free region (NFR) or no nucleosome-free (other) region for Q, W and D. The mean of either distribution is shown by a solid black line.

In order to comprehensively determine the accessible chromatin landscape in the *A. mellifera* genome, we profiled biological replicate pools of five brains from newly-eclosed adult queen (Q), worker (W) and male drone (D) central brains by high-coverage ATAC-seq. The brain is critical to understand how the three distinct behavioural polyphenisms are primed at adult emergence and newly-eclosed adult honey bees were selected in order to avoid confounding factors of differential age and/or before functional specialisations and activity-induced biological changes. Replicates show a very strong and significant correlation across all phenotypes (Supplementary Figure S1; $\rho >0.91$; *P*-value < $2.2 \times {10}^{ - 16})$ and ATAC-seq fragment size distribution shows that a large proportion of reads are < 100 bp, which represents the nucleosome-free regions (NFRs; Figure 1A). The fragment distribution also shows a clear periodicity that is indicative of nucleosome occupancy. In order to separate NFRs and mono-nucleosomes, we used ATAC-seq reads in the range of (0–100 bp, NFR; 150–247 bp, mono-nucleosomes; 315–473 bp di-nucleosomes) (30). We find enrichment of NFRs around the transcriptional start sites (TSS) of genes in Q, W and D, and a well-positioned nucleosome downstream (Figure 1B). Overall, the majority of genes in the honey bee genome contain at least one NFR (Q: 66.3%; W: 71.9%; D: 70.2%) predominantly located within intronic regions (> 40% of all ATAC-seq peaks in Q, W or D), with the remainder mainly within promoter (> 15%) and 5’ UTR regions (> 20%; Figure 1C). Since NFRs are mostly localised close to or within genes, we next investigated the correlation with transcription. We profiled gene expression in matched newly-eclosed adult Q, W and D central brains by RNA-seq and replicates show a very strong and significant correlation across all phenotypes (Supplementary Figure S1; $\rho >0.86;$*P*-value < $2.2\ \times {10}^{ - 16}).$ In all cases, genes containing at least one NFR show a significant increase in expression (*P*-value < $2.2\ \times {10}^{ - 16})$ compared to those genes that do not contain a NFR (Figure 1D and Supplementary Figure S2). Therefore, in queen, worker and the male drone, the majority of NFRs are associated with actively transcribed regions and may have the potential to regulate adult phenotype-specific gene expression.

### Intronic regions in *A. mellifera* are associated with chromatin states characteristic of *cis*-acting DNA regulatory elements

**Figure 2.**
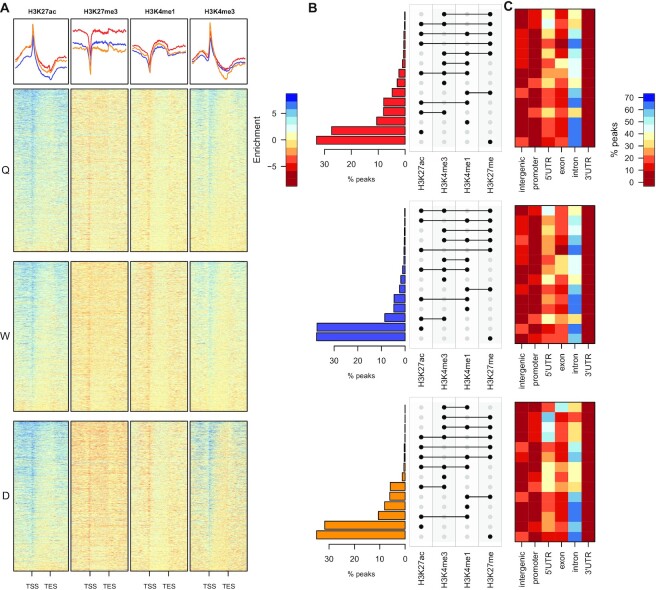
Defining chromatin states in *A. mellifera* queen, worker and drone. (**A**) Heatmaps showing the distribution of H3K4me1, H3K4me3, H3K27ac and H3K27me3 across genes in Q, W and D. Blue indicates enriched and red, depleted. Each row is -2 kbp to TSS, TSS to TES and TES to +2 kbp. Genes are ordered by read enrichment across all histone modifications. (**B**) UpSet plot showing the overlap of H3K4me1, H3K4me3, H3K27ac and H3K27me3 in Q, W and D to define different chromatin states. The vertical bar plot represents the percentage of genomic regions, and the dot plot shows the histone modification(s) present in that state. (**C**) Heatmaps showing the enrichment of chromatin states in annotated genomic features in Q, W and D. Dark blue indicates high enrichment, dark red indicates low enrichment and pale yellow, no enrichment.

We next wanted to determine the profile of histone post-translational modifications (PTMs) that are characteristic of *cis*-acting DNA regulatory elements in other organisms. Therefore, we profiled biological replicate pools of matched newly-eclosed adult Q, W and D central brains by ChIP-seq (five brains per pool) and determined the genome-wide distribution of four histone PTMs: H3K4me1, H3K4me3, H3K27ac, H3K27me3 (32–35). Replicates show a very strong and significant correlation across all histone PTMs and adult phenotypes (Supplementary Figure S1; $\rho >0.8$; *P*-value < $2.2\ \times {10}^{ - 16}).$ We find enrichment of H3K4me3 and H3K27ac, and depletion of H3K4me1 and H3K27me3 around the TSSs of genes in Q, W and D (Figure 2A). H3K4me1 is mostly enriched over gene bodies (Figure 2A). Furthermore, we identify thousands of queen, worker and drone -specific distributions for all four histone PTMs (Supplemental Figure S3). Of the 23,458 transcripts in the honey bee genome, 54.9% in Q, 57.8% in W and 56.7% in D, show high levels of enrichment (> 3-fold over input) for at least one of the four histone PTMs profiled. Next, in order to examine the chromatin state distribution over annotated genomic features more closely, we wanted to determine the degree of overlap between these histone PTMs. In the overwhelming majority of cases, all four histone PTMs alone or in combination, are significantly more enriched in intronic regions than other genomic locations in Q, W and D (Figure 2B and C). In all cases, H3K27ac and H3K27me3 alone, are the most abundant chromatin states found in introns, followed by intersections of H3K4me3/H3K27ac, H3K4me1/H3K27ac and H3K4me1/H3K27me3. In fact, H3K4me3 is co-localised with H3K27ac significantly more often than being enriched alone (*P*-value: $ <\, {10}^{-16}$ for all phenotypes Fisher test odds ratio: > 20), however, in both cases, these two chromatin states are more equally distributed between 5’ UTR and intronic regions than any other. Interestingly, the male drone shows one significant difference from the two female castes. In contrast to Q and W, the most abundant intronic combinatorial chromatin state are those regions marked by both H3K4me1 and H3K27ac (Figure 2B and C). Finally, and in all cases, intergenic regions show the highest enrichment for chromatin states containing H3K27me3. Therefore, taken together, intronic regions and to a lesser extent, 5’ UTRs, in Q, W and D are significantly enriched in chromatin states that are associated with *cis*-acting DNA regulatory elements.

### Chromatin accessibility is associated with H3K27ac, H3K4me3/H3K27ac and specific transcription factor binding motifs

**Figure 3.**
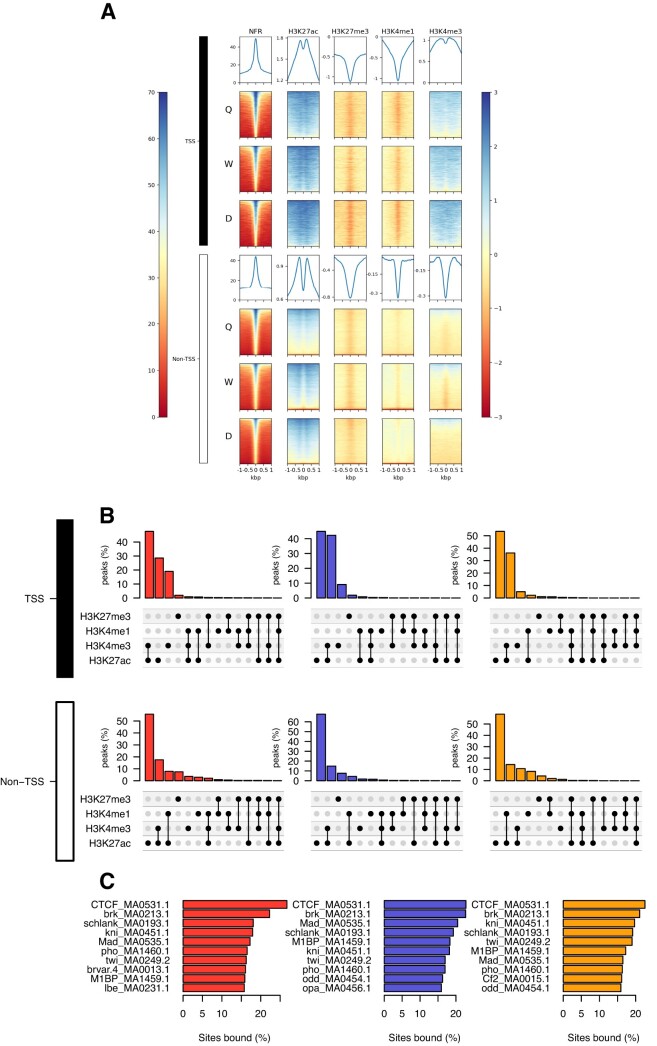
Nucleosome-free regions are associated with H3K27ac, H3K4me3 and transcription factor occupancy. (**A**) Heatmap of ATAC NFR, H3K4me1, H3K4me3, H3K27ac and H3K27me3 read enrichments for ATAC-seq NFR peaks around the TSS (upper) and non-TSS (lower). Left colour scale bar represents the enrichment in ATAC NFR and the right colour scale bar represents enrichment for all ChIP-seq data. (**B**) UpSet plot showing the overlap of H3K4me1, H3K4me3, H3K27ac and H3K27me3 with TSS (upper) and non-TSS (lower) NFRs in Q, W and D. The vertical bar plot represents the percentage of genomic regions, and the dot plot shows the histone modification(s) present. (**C**) Bar plot showing the top 10 most occupied transcription factor binding sites within NFRs for each caste based on TOBIAS footprinting analysis.

To determine *cis*-acting DNA regulatory elements in *A. mellifera*, we sought to integrate chromatin states with nucleosome-free regions. Given the association of H3K4me3 with TSSs, we separated the chromatin states for those within 200 bp upstream (promoter) and downstream (5’ UTR) of annotated TSSs and everything else (non-TSS) (Figure 3A). In Q, W and D, both TSS and non-TSS NFRs, are significantly enriched in flanking nucleosomes enriched for H3K27ac and depleted for H3K27me3 (Figure 3A). However, in stark contrast to non-TSS associated NFRs, TSS-associated NFRs are also strongly enriched with intersections of H3K4me3/H3K27ac or H3K4me3 alone (Figure 3B). Overall, we find that H3K4me3, H3K27ac and H3K4me3/H3K27ac are associated with > 70% of all TSS accessible chromatin regions (Q: 73.4%; W: 71.7%; D: 70.1%; Figure 3B), with intersections of H3K4me3/H3K27ac accounting for > 25% of all TSS NFRs (Q: 36%; W: 31%; D: 26%; Figure 3B). However, for non-TSS associated NFRs, overwhelmingly we identify H3K27ac alone, a marker of active enhancers in other organisms (32–35), as the most predominant chromatin state (Q: 14.3%; W: 10.5%; D: 11.4%; Figure 3B). For non-TSS accessible chromatin regions, the intersection of H3K4me3/H3K27ac accounts for only < 10% of all NFRs (Q: 9.7%; W: 7.1%; D: 7.7%; Figure 3B). In contrast to Q and W, in the male drone, the next most abundant chromatin state after H3K27ac alone are intersections of H3K4me1/H3K27ac, accounting for 6.1% of all NFRs (Q: 3.3%; W: 1.8%; *P*-value: $ <\, {10}^{ - 16}$ Fisher test odds ratio: 9.33; Figure 3B). These regions are located near genes with GO terms associated with signal transduction (GO:0007165) and protein tyrosine kinase activity (GO:0004713; Supplementary Figure S4A). Significantly, additional chromatin states characteristic of active (H3K4me1/H3K27ac), mixed (H3K4me1/H3K27ac/H3K27me3), primed (H3K4me1), poised (H3K4me1/H3K27me3) and repressed (H3K27me3) enhancer elements in other organisms (32–35) were also more abundant in non-TSS accessible chromatin regions, accounting for 7.3% (Q), 3.3% (W) and 12.9% (D) of all non-TSS NFRs in the honey bee (< 1% of TSS-associated NFRs; see Figure 3B).

Thus far, our data demonstrates that NFRs are enriched with chromatin states associated with CREs, so next we wanted to determine what factors could activate these regions in honey bees. Due to the absence of available antibodies that cross-react with honey bee specific DNA-binding proteins, we performed footprinting analysis of the ATAC-seq data using TOBIAS (Transcription factor Occupancy prediction By Investigation of ATAC-seq Signal) (41) in order to determine any transcription factor binding within these accessible chromatin regions. In Q, W and D, we find visible footprints of TF binding matched to known insect TFBS across NFRs (Q: 67,764; W: 63,431; D: 69,323 and Supplemental Figure S5). We observe significant conservation of the top 10 most occupied TF binding sites across Q, W and D NFRs, with DNA motifs for Brinker (brk) and the insulator associated protein CCCTC-binding factor (CTCF) showing greatest occupancy in all adult phenotypes (> 20%; Figure 3C). Interestingly however, we also observe phenotype-specific TF occupancy with Broad (br(var.4)) and Ladybird early (Ibe) binding sites with > 15% occupancy in Q only, and > 15% of Odd paired (opa) and > 15% of Chorion factor 2 (Cf2) binding site occupancy only in W and D respectively (see Figure 3C). In addition, the relatively abundant drone intronic regions marked by H3K4me1/H3K27ac reveal enrichment for occupied TFBSs associated with the visual system in *Drosophila melanogaster*, such as BarH1/BarH2 (B-H1/B-H2) (48) and Visual system homeobox 2 (Vsx2) (49) (Supplementary Figure S4B), supportive of a role for these drone-specific CREs in eye function, a key morphological distinction between male and female honey bees (10). Taken together, in Q, W and D newly-eclosed adult honey bees, the overwhelming majority of non-TSS nucleosome-free regions have all the molecular hallmarks of *cis*-acting DNA regulatory elements such as enhancers and insulators.

### Queen, worker and drone -specific chromatin accessibility highlights phenotype-specific DNA sequence motifs and transcription factor binding

**Figure 4.**
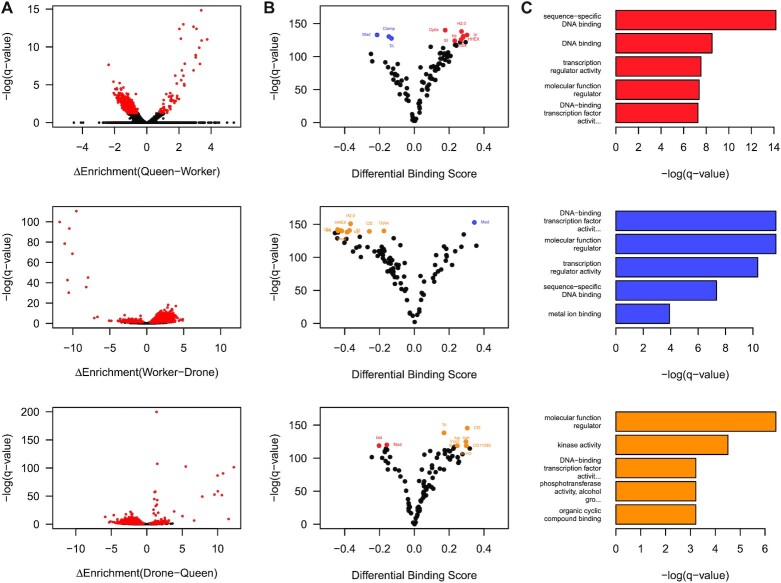
Phenotype-specific nucleosome-free regions are associated with differential transcription factor occupancy. (**A**) Volcano plots of the differences in enrichment of ATAC-seq between queen and worker (top panel), worker and drone (middle panel) and drone and queen (bottom panel) against the negative log *q*-value. In black are regions which fall below the genome wide threshold of significance (FDR > 0.05). In red are those regions which reach genome wide significance (FDR ≤ 0.05). (**B**) Volcano plots of the differential transcription factor binding score queen and worker (top panel), worker and drone (middle panel) and drone and queen (bottom panel) against the negative log *P*-value. Highlighted are the top ten transcription factors by *P*-value in each comparison. (**C**) The negative log *P*-value for the top five molecular function GO terms for those genes that show differential transcription factor occupancy in queen (top), worker (middle) and drone (bottom).

Having established that in Q, W and D honey bees, NFRs are predominantly flanked by H3K27ac modified nucleosomes, and harbour occupied TFBSs, we next wanted to determine any phenotype-specific differences. Pairwise comparison of all ATAC-seq data between Q, W and D reveal significantly different patterns of accessibility (Figure 4A). For Q versus W, we identified 59 unique regions in Q and 851 in W (FDR < 0.05). For W versus D, we identify 9252 unique regions in W and 302 in D. Finally, for D versus Q, we identify 300 unique regions in D and 4382 in Q. Next, we wanted to link these differences to differentially occupied TFBSs. Pairwise comparison of differential footprinting data, indicates that DNA motifs for Mothers against dpp (Mad), Trithorax-like (Trl) and Chromatin-linked adaptor for MSL proteins (Clamp) are significantly more occupied (*P*-value < 0.05) in W than Q, and those including Haematopoietically expressed homeobox (HHEX), Dbx (Dbx) and Broad (br) more in Q than W (Figure 4B). For W versus D, DNA motifs for Mad are significantly bound more in W than D, and those including Visual system homeobox 2 (Vsx2) and Ultrabithorax (Ubx) in D. Finally, in D versus Q, DNA motifs including those for Brain-specific homeobox (Bsh), BarH2 (B-H2) and Chorion factor 2 (Cf2) are significantly more occupied in D than Q, with motifs for Mad and Buttonhead (btd) more bound in Q (Figure 4B). Gene ontology analysis of genes that harbour significant phenotype-specific differential TFBS occupancy reveal a significant association with transcriptional regulation, with the majority encoding further TFs (Figure 4C). In Q, W and D overrepresented GO terms (*P*-value < $1.6\ \times {10}^{ - 5})$ significantly associate with the molecular functions of sequence-specific DNA binding (GO:0043565), DNA-binding transcription factor activity (GO:0003700) and DNA binding (GO:0003677). In contrast, molecular function regulator activity (GO:0098772) and kinase activity (GO:0016301) are only significantly enriched in male drones (Figure 4C). Thus, specific DNA sequence motifs and TFBS occupancy are associated with phenotype-specific nucleosome-free regions in *A. mellifera* and many of these are within genes that encode further transcriptional regulators.

### Transcription factor gene networks reveal queen, worker and drone -specific expression of regulatory genes in *A. mellifera*

**Figure 5.**
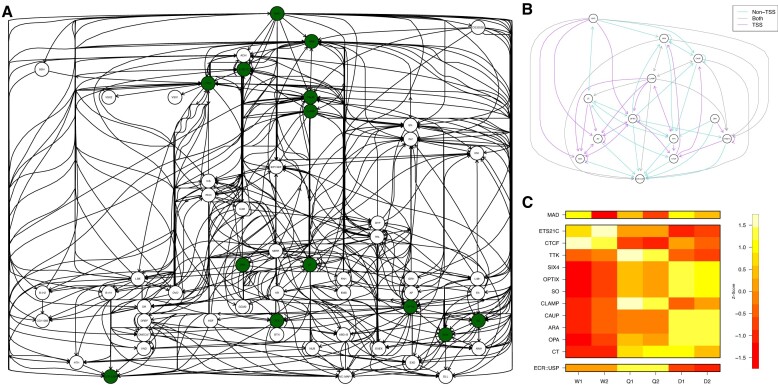
Mad transcription factor networks reveal phenotype-specific expression of downstream target transcription factor genes in *A. mellifera*. (**A**) The combined TF–TF network built of all TFBS with TF occupancy in promoters from Q, W and D starting from Mad transcription factor. Each directed edge indicates binding site occupancy of that TF in the respective gene promoter of the other TF, found by the TOBIAS CreativeNetwork module. Coloured circles indicate TFs shown in (B) and (C). (**B**) Reduced TF–TF subnetwork of (A) in which TFs downstream of Mad show differential expression between the three phenotypes. Directed edges indicate binding site occupancy in the respective gene promoter found by the TOBIAS CreativeNetwork module. Coloured arrows indicate whether binding of the TF is either located within the TSS region; mint (defined here as –2 kb to +200 bp), non-TSS regions; purple (e.g. any other location within the gene) or a combination of the two; grey. (**C**) A heat map of the expression levels of each of the TFs across all three phenotypes broken out based on the levels of the transcriptional network in (B). Each row is a *z*-score of the log expression value and duplicates are shown.

Since we observe abundant and differential occupancy of Mad TF binding motifs in each of the Q, W and D pairwise comparisons and that the majority of these reside in genes encoding further TFs (Figure 4B and Supplemental Figure S6), we wanted to determine how these TF activities may connect. We applied the TOBIAS network module to these TF targets in order to uncover a TF–TF gene regulatory network in Q, W and D based on ATAC-seq footprints. The resulting gene regulatory network includes 55 TF nodes and 478 directed edges (Figure 5A). Strikingly, by focussing on just a subset of these in each adult phenotype (13 nodes), we show that while *A. mellifera Mad* itself shows little differential expression between Q, W and D, the downstream TF cascade that results from differential Mad TFBS occupancy does show phenotype-specific expression patterns (Figure 5B and C). In particular, six immediate downstream Mad TF target genes: *araucan* (*ara*), *caupolican* (*caup*), *cut* (*ct*), *Optix* (*Optix*), sine *oculis* (*so*) and *Six4* (*Six4*) show a decreased pattern of expression in W relative to Q and D (Figure 5C). In stark contrast, the male drone shows a significant increase in expression of these six immediate downstream Mad TF targets relative to the female worker, which in the case of *so* and *Optix*, both driven through promoter binding, most likely relates to the much larger eyes and optic lobes in honey bee drones (10). Finally, the female queen has intermediate levels of expression of these same genes relative to the worker and drone (Figure 5C). Of these six immediate downstream Mad TF target genes, *ct* shows the most significant W-specific decreased expression pattern, linked to non-promoter binding, as do four further downstream targets of Cut: *tramtrak* (*ttk*) relative to Q, also linked to non-promoter binding, and *Six4*, *so* and *Optix* relative to D linked to promoter binding (Figure 5C). Conversely, the gene encoding the insulator associated CCCTC-binding factor, *CTCF*, a downstream target of Cut in our network, shows significantly increased W-specific expression, and relative to Q and D, intermediate expression of the CTCF target genes *ecdysone receptor* (*EcR*) and *ultraspiracle* (*usp*) (*EcR::USP*); the end point of the nodule of genes and two crucial insect nuclear receptors (homologs of vertebrate Farnesoid X and Retinoid X Receptors (50,51); Figure 5C). Conversely, in both queen and drone, *ct* has significantly increased expression and *CTCF* significantly decreased expression relative to the worker, with increased relative expression of *EcR* and *USP* in queen and decreased expression in drone. High expression of *EcR* and *usp* in queen may be accounted for by the relatively queen-specific expression of *ttk* and *Chromatin-linked adapter for MSL proteins* (*Clamp*), TFs that can act upstream and independently of CTCF (Figure 5B). Notably, TTK and CLAMP are connected to each other exclusively through TSS binding. In summary, by analysing the TF:TF network associated with differential Mad DNA motif occupancy within nucleosome-free regions, we reveal a previously unknown pathway, that is associated with a cascade of queen, worker and drone -specific expression patterns of downstream regulatory target genes that have the potential to contribute to polyphenism in *A. mellifera*.

## DISCUSSION

Here, we present the first genome-wide high-resolution chromatin accessibility profiles for each distinct adult honey bee phenotype. Using an multiomic approach that combines ATAC-seq, ChIP-seq and RNA-seq, we identify for the first time thousands of nucleosome-free regions throughout the honey bee genome harbouring *cis*-acting DNA regulatory elements that are defined by H3K27ac chromatin states, transcription factor occupancy and associated gene expression. Importantly, we identify numerous queen, worker and drone-specific differences allowing the first comparative analysis of chromatin structure and DNA regulatory sequences between *Hymenoptera* male and female polyphenisms and also the first between *Hymenoptera* female castes that show a reproductive division of labour. Finally, we elucidate phenotype-specific gene expression pathways based on transcription factor occupancy data and present previously unknown regulatory networks that may underpin the molecular basis of polyphenism in the honey bee.

Our multiomic analysis is focussed specifically on all three honey bee polyphenisms at adult eclosure, allowing us to obtain an overview of distinct brain epigenomes and chromatin accessibility at the endpoint of development and the beginning of adult behavioural plasticity. Therefore, the brain is the critical organ to study in order to understand how the three distinct behaviours are primed at adult emergence. We identify thousands of nucleosome-free regions throughout the genomes of queen (*n* = 22,656), worker (*n* = 29,688) and male drone (*n* = 27,536), which are distributed mainly within intragenic regions. Significantly, unlike mammalian genomes, we observe very few distal NFRs (< 5%), indicating a close coupling between accessible regions and genes, similar to that reported in other Arthropods (52–54). This is further supported by the correlation between mRNA abundance and presence of an NFR within the gene, suggesting possible functional interactions between these regions and the nearby gene. Overwhelmingly, the most prevalent chromatin states associated with nucleosome-free regions in all three honey bee polyphenisms are H3K4me3, H3K27ac and intersections of H3K4me3/H3K27ac (> 44% of all NFRs). Interestingly, these three chromatin states robustly delineate TSS and non-TSS NFRs and correlate with underlying gene expression. NFRs outside the vicinity of the TSS are overwhelmingly characterised by an intronic location and flanking H3K27ac alone modified nucleosomes and at a lower frequency, various additional combinations of H3K4me1, H3K27ac and H3K27me3 chromatin states. Taken together, many of these non-TSS NFRs have chromatin states characteristic of active, neutral, poised and/or repressed *cis*-regulatory elements, such as enhancer regions (32–35) and is consistent with our previous studies during larval development, where we found that intronic H3K27ac robustly defined the queen and worker caste (20). We do observe a small but significant number of non-TSS NFRs that are characterised by H3K4me3/H3K27ac chromatin states, which may represent previously unannotated alternative or novel TSSs rather than active enhancers. Additionally, relative to queen/worker, throughout our analyses we observe that the greatest separation is between haploid male and diploid female adult phenotypes, suggesting that in addition to the genetic-determination of sex (55), chromatin-based mechanisms may also play an important role, possibly in the maintenance of the sex-determined state. This is supported by the fact that drones also have a distinct diet (both quantity and quality), strongly implying that other non-genetic factors, which likely operate via epigenomic mechanisms, are involved in determining the drone polyphenism (6,8,11).

To our knowledge only one previous study, over a decade ago, has reported the genome-wide location of a TF within the honey bee genome (56). Instead, studies have focussed on motif enrichment analyses, irrespective of occupancy and therefore also of uncertain functional significance (20,23,56,57). Here, we report the utility of ATAC-seq footprinting (41) on high-coverage genomic data in determining DNA-binding site occupancy genome-wide across all three adult honey bee polyphenisms, allowing us to circumvent a lack of specific antibody-based molecular tools, often associated with studying emergent model organisms. We find extensive TF occupancy across each polyphenism and unsurprisingly, we observe similar levels of occupancy and the same over-represented top 10 most bound motifs across all three adult polyphenisms, indicating commonality in the TF networks associated with fundamental honey bee genome regulation. The presence of abundantly occupied CTCF-motifs is strongly suggestive of insulator DNA elements at these NFRs (30). In addition, we also observe high occupancy of BEAF32 and Su(H)w motifs within NFRs (> 9% and > 13% of all motifs, across all phenotypes), two further characterised insulator binding proteins in *Drosophila melanogaster*, indicating that a significant proportion of NFRs identified in this study are insulator elements (58) (Q: 10.3%; W: 6.9%; D: 7.3%). The remainder of the most highly occupied DNA motifs within NFRs are TFBSs associated with DNA-binding proteins that have been demonstrated to bind enhancers in order to regulate gene expression and we observe significant phenotype-specific distribution that reflect differential TFBS occupancy. Strikingly, many of these differential NFRs are within genes encoding further transcription factors. For example, we observe extensive but differential binding between queen, worker and drone at DNA motifs for Mothers against dpp (Mad, a SMAD family member in vertebrates), an enhancer-associated transcriptional activator that mediates the bone morphogenetic protein (BMP) signalling cascade, acting downstream of Decapentaplegic (Dpp) (59). This is consistent with our previous studies that demonstrated enrichment of Mad motifs within intronic regions of workers during larval development (20). In other organisms, Mad has been demonstrated to interact directly with CREB-binding protein (CBP) (60), co-localize with regions of H3K27ac at enhancers (61) and affect gene expression in a CBP-dependent manner (62). Analysis of the TF binding network of Mad motif occupancy reveals a previously unknown cascade of phenotype-specific expression of downstream target TFs, converging on the genes encoding two crucial insect nuclear receptors; *ecdysone receptor* and *ultraspiracle* (insect homologs of the Farnesoid X and Retinoid X Receptors (50,51)). EcR and USP have been studied extensively in *Drosophila melanogaster*, where functional heterodimers are formed in order to mediate important tissue-specific transcriptional responses to two crucial steroid hormones, ecdysone and Juvenile hormone (JH), during major developmental transitions such as larval-larval molting and larva-pupal-adult metamorphosis, and in female reproductive maturation (63,64). Evidence of a role for nuclear hormone receptors, JH and ecdysone in caste differentiation comes mainly from studies in ants and bees. In the ant *Harpegnathos saltator*, EcR and an additional nuclear receptor, Methoprene-tolerant, have been demonstrated to mediate the response to JH and ecdysone in driving caste-specific transcription and behaviour (65). In *A. mellifera*, EcR and USP have been suggested to have important roles in queen/worker caste differentiation (23), pupation (66,67) and in the regulation of specific behaviours in adult worker honey bees (56,57). Our upstream hierarchical TF network analysis suggests that an additional morphogen, Dpp, acting through Mad TFBSs, could be important in establishing phenotype-specific thresholds of *EcR* and *USP* expression in *A. mellifera*. In newly-eclosed adults, this may be important in order to enable the integration of polyphenism-specific titres of JH and ecdysone into specific transcriptional programmes. Differing titres of JH and ecdysone have been reported in many social insect castes and in honey bees, during both pre-pupal development (68) and adult maturation (69–72). Further experiments such as RNAi knockdown are required to manipulate levels of Dpp in each polyphenism, in order to determine exactly what, if any, role Dpp has in honey bee polyphenism.

In summary, our work reveals the regulatory genome of the honey bee across all three adult polyphenisms. For the first time, we report significant chromatin-based sex-specific differences and uncover a potential regulatory mechanism focused around the previously reported role of Mad in *A. mellifera*, which leads to the targeting of two key nuclear receptors involved in caste-determination and adult behavioural maturation, ecdysone receptor and ultraspiracle, and therefore ultimately, in influencing downstream transcriptional programmes involved in honey bee polyphenism.

## Supplementary Material

gkac992_Supplemental_File

## Data Availability

The dataset(s) supporting the conclusions of this article are available in the National Centre for Biotechnology Information (NCBI) Gene Expression Omnibus (GEO) repository, under accession number GSE206995. [https://www.ncbi.nlm.nih.gov/geo/query/acc.cgi?acc=GSE206995] and available online to view using Integrated Genomics Viewer: https://tinyurl.com/22n9kdj8.

## Notes

Present address: Robert Lowe, Altos Labs, Cambridge Institute of Science, Portway Building, Granta Park, Great Abington, Cambridge CB21 6GP, UK.

Present address: Marek Wojciechowski, International Institute of Molecular and Cell Biology in Warsaw, 4 Ks. Trojdena Street, 01-109 Warsaw, Poland.
