# Correction to ‘Chromatin accessibility-based characterisation of brain gene regulatory networks in three distinct honey bee polyphenisms’

**DOI:** 10.1093/nar/gkad883

**Published:** 2023-10-09

*Nucleic Acids Research*, Volume 50, Issue 20, 11 November 2022, Pages 11550–11562, https://doi.org/10.1093/nar/gkac992

The originally published version of this manuscript required correction.

In Figure 3A, top heat map panel was omitted.


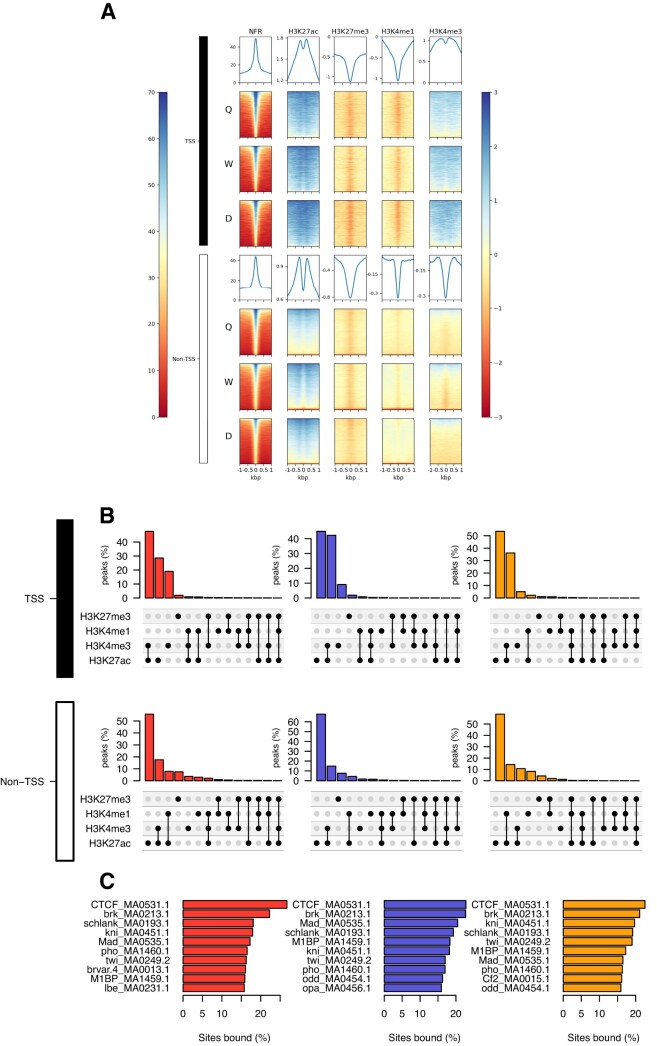


In Figure 5C, the TTK gene row was duplicated.


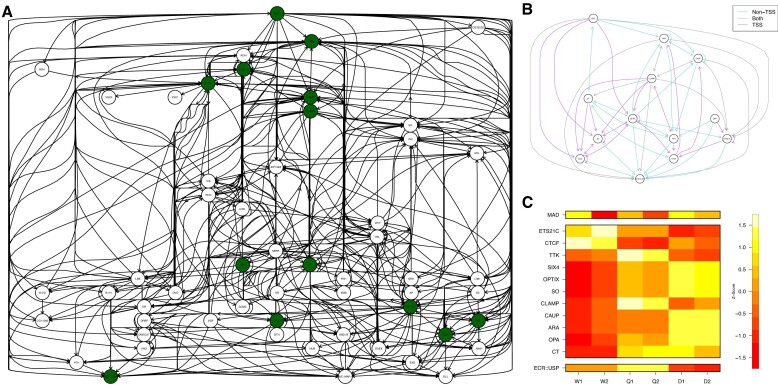


These errors have been corrected.

