# Supplementary material for: Chromatin accessibility-based characterisation of brain gene regulatory networks in three distinct honey bee polyphenisms
Source: Nucleic Acids Res. 2022 Nov 4;50(20):11550–62. doi: 10.1093/nar/gkac992 (PMC9723623; doi:10.1093/nar/gkac992)
Supplement: gkac992_Supplemental_File [file gkac992_supplemental_file.pdf]

Supplementary Information

Chromatin Accessibility-Based Characterization of Brain Gene Regulatory Networks in Three Distinct Honey Bee Polyphenisms.

Robert Lowe,[1,3,5] Marek Wojciechowski,[2,4] Nancy Ellis,[2] and Paul J. Hurd[2,5]

[1] RER Consultants, 28 Worbeck Road, London, SE20 7SW, UK.

[2] School of Biological and Behavioural Sciences, Queen Mary University of London, Mile End Road, London, E1 4NS, UK.

[3] Current address: Altos Labs, Cambridge, UK.

[4] Current address: International Institute of Molecular and Cell Biology in Warsaw, 4 Ks. Trojdena Street, 01-109 Warsaw, Poland.

The authors wish it to be known that, in their opinion, the first two authors should be regarded as joint First Authors.

[5] Corresponding authors

E-mail: p.j.hurd@qmul.ac.uk

E-mail: rlowe@altoslabs.com

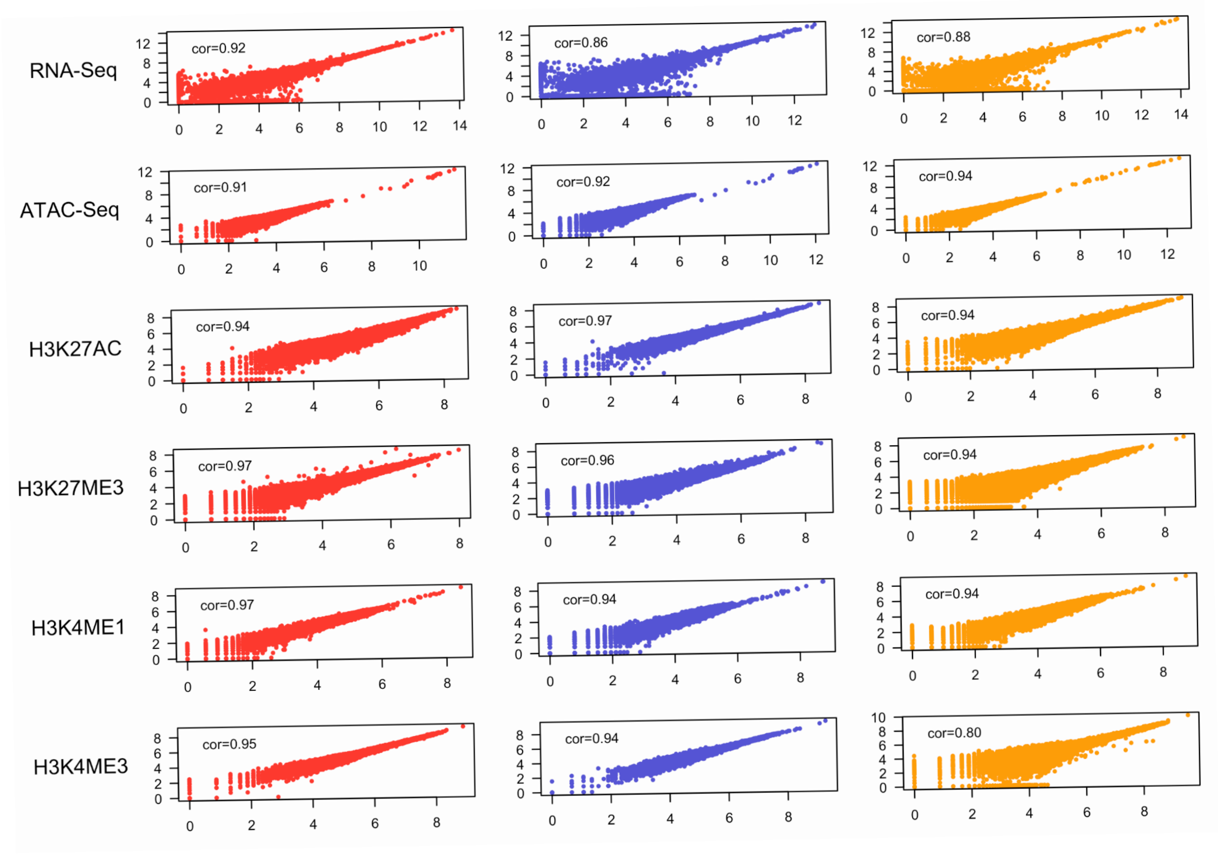

Supplemental Figure S1. Scatter plots of the log of the counts for each of the replicate experiments. Replicate 1 on the x-axis, replicate 2 on the y-axis. For RNA-Seq, this is the number of reads within each mRNA, while for ATAC-seq/ChIP-seq experiments, these are the counts in the called peakset from DiffBind used for all analyses. Queen (red), worker (blue) and drone (orange).

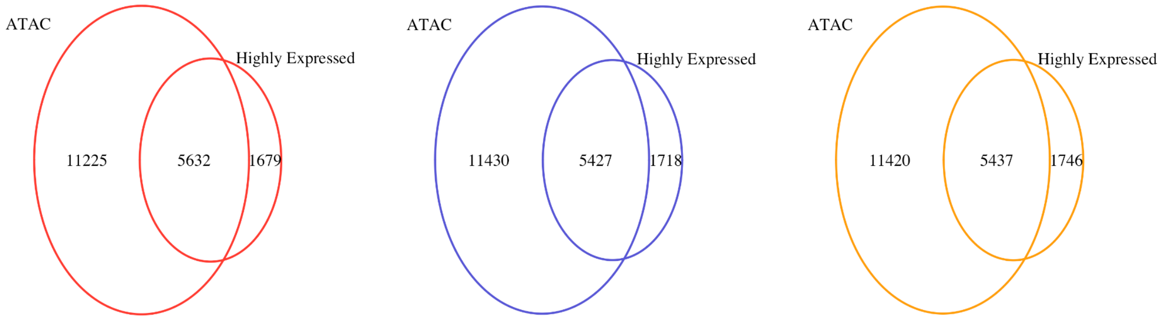

Supplemental Figure S2. Venn diagram showing the overlap of highly expressed genes (log(counts+1) > 5) and genes with an ATAC-seq peak for each of the three adult honey bee phenotypes (queen: red; worker: blue; drone: orange).

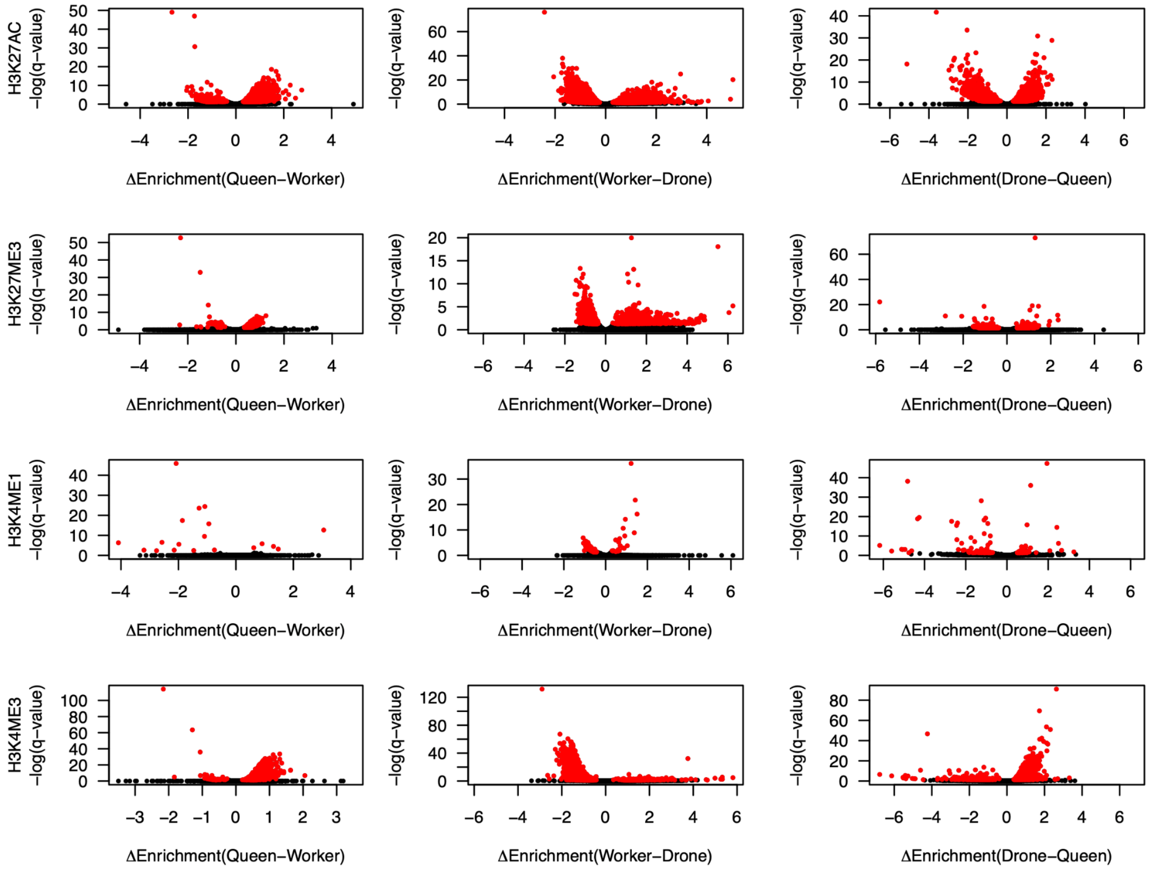

Supplemental Figure S3. Volcano plots of the difference in enrichment between queen, worker and drone against the negative log p-value for H3K4me1, H3K4me3, H3K27ac and H3K27me3. In black are regions which fall below the genome wide threshold of significance (FDR > 0.05). In red are those regions which reach genome wide significance (FDR < 0.05) and have a larger than 3-fold difference in enrichment above input between adult phenotypes.

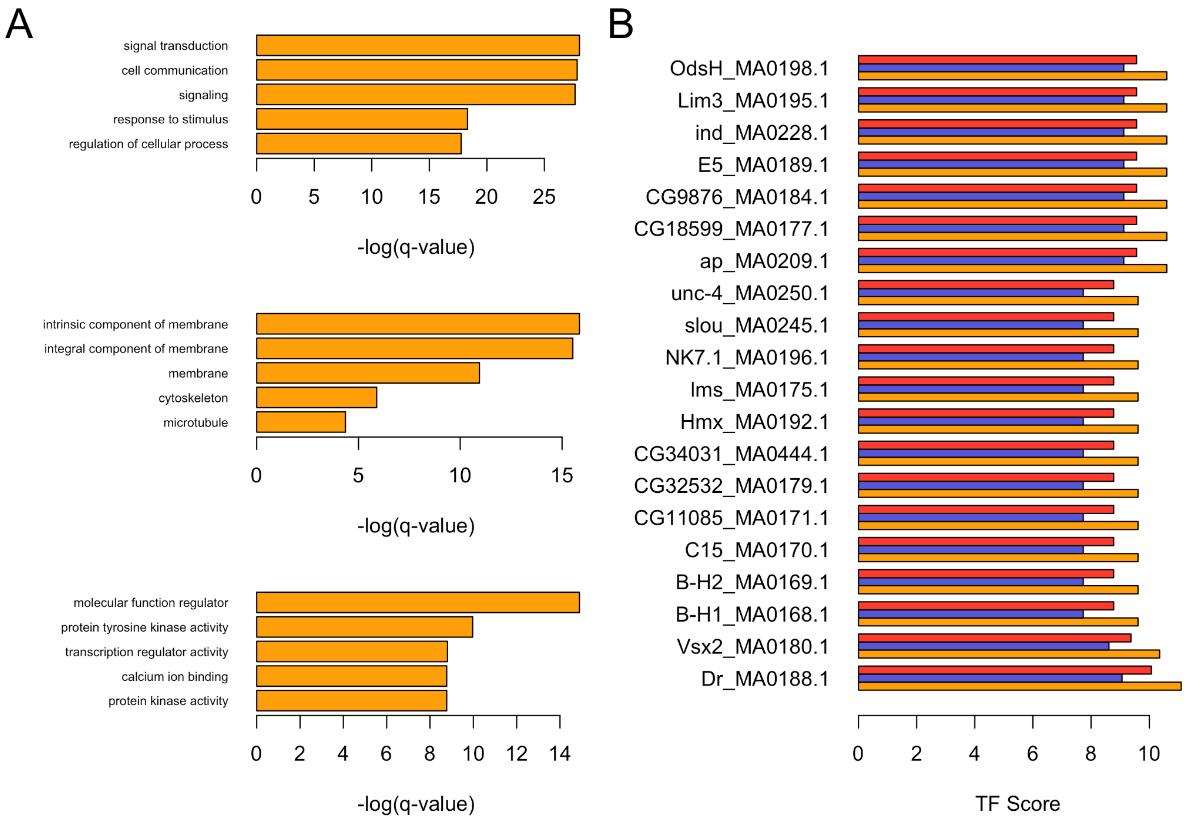

Supplemental Figure S4. (A) The negative log q-value for the top five molecular GO terms in each of the categories (Biological Processes (top), Cellular Components (middle) and Molecular Functions (bottom)) for those intragenic regions that are marked by H3K4me1/H3K27ac in the male drone. (B) The mean of footprint scores for all TF binding for each motif as reported by TOBIAS for the top 20 TFs which show the largest difference in score between drone (orange) versus queen (red) or worker (blue). The TF score was calculated for TF binding in genic regions marked by H3K4me1/H3K27ac.

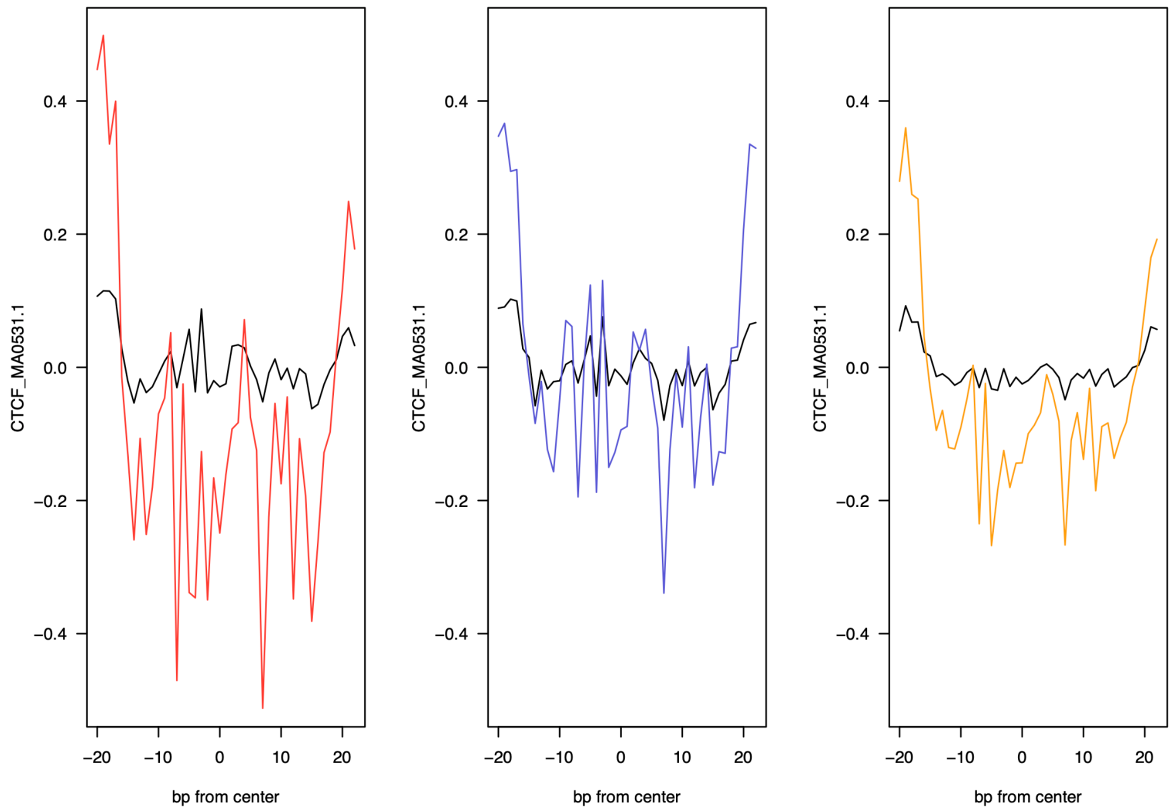

Supplemental Figure S5. CTCF-occupied DNA binding sites were identified using computational matching between the PWM and the reference sequence using the TOBIAS pipeline. Shown in red is enrichment for occupied sites (coloured line) vs unbound CTCF sites (black) in queen (red), worker (blue) and drone (orange).

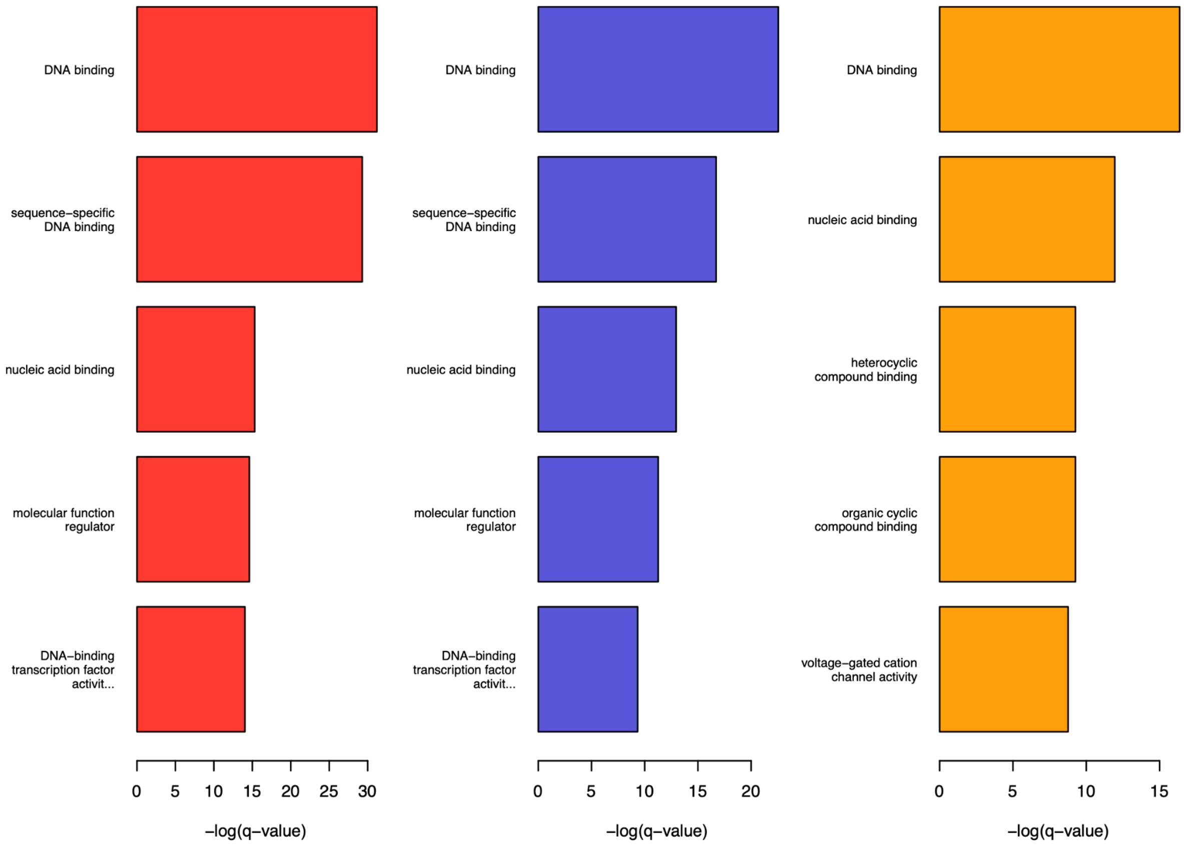

Supplemental Figure S6. The negative log q-value for the top five molecular function GO terms for those genes that show differential Mad transcription factor motif occupancy in queen (red), worker (blue) and drone (orange).

Adult Type	Biological Replicate	Experiment	Read Number	Mapped Reads
Queen	1	ATAC-seq	133,621,280	66,365,814
Queen	2	ATAC-seq	96,567,668	48,033,580
Worker	1	ATAC-seq	104,405,184	51,972,019
Worker	2	ATAC-seq	96,625,044	48,058,344
Drone	1	ATAC-seq	170,164,020	84,518,453
Drone	2	ATAC-seq	137,453,096	68,270,987
Queen	1	ChIP-seq H3K27ac	91,905,168	45,259,952
Queen	2	ChIP-seq H3K27ac	83,262,092	40,542,727
Queen	1	ChIP-seq H3K27me3	100,706,868	48,082,824
Queen	2	ChIP-seq H3K27me3	88,497,708	40,945,654
Queen	1	ChIP-seq H3K4me1	144,436,508	71,860,308
Queen	2	ChIP-seq H3K4me1	131,732,000	65,523,446
Queen	1	ChIP-seq H3K4me3	84,775,820	40,731,932
Queen	2	ChIP-seq H3K4me3	127,663,540	61,682,931
Queen	1	Input sample	80,398,600	39,943,164
Worker	1	ChIP-seq H3K27ac	132,377,036	65,859,107
Worker	2	ChIP-seq H3K27ac	121,392,292	60,442,787
Worker	1	ChIP-seq H3K27me3	85,516,772	42,467,080
Worker	2	ChIP-seq H3K27me3	88,994,884	43,987,657
Worker	1	ChIP-seq H3K4me1	92,787,720	46,221,259
Worker	2	ChIP-seq H3K4me1	96,531,284	48,072,991
Worker	1	ChIP-seq H3K4me3	96,879,536	40,821,296
Worker	2	ChIP-seq H3K4me3	121,148,704	59,876,000
Worker	1	Input sample	106,356,944	52,919,025
Drone	1	ChIP-seq H3K27ac	138,461,496	68,432,713
Drone	2	ChIP-seq H3K27ac	104,284,776	51,589,664
Drone	1	ChIP-seq H3K27me3	159,739,172	78,674,774
Drone	2	ChIP-seq H3K27me3	94,788,556	46,306,142
Drone	1	ChIP-seq H3K4me1	129,367,060	64,297,616
Drone	2	ChIP-seq H3K4me1	134,797,236	67,005,023
Drone	1	ChIP-seq H3K4me3	92,777,636	47,194,411
Drone	2	ChIP-seq H3K4me3	167,056,764	78,920,437
Drone	1	Input sample	239,181,548	118,791,230
Queen	1	RNA-seq	100,253,928	44,373,789
Queen	2	RNA-seq	48,315,950	21,203,822
Worker	1	RNA-seq	28,817,646	12,432,183
Worker	2	RNA-seq	17,547,489	7,685,185
Drone	1	RNA-seq	90,912,241	40,453,916
Drone	2	RNA-seq	26,538,438	11,287,495

Supplemental Table S1. Sequencing and Mapping Statistics.
